# Comparative outcomes of left atrial appendage occlusion with and without catheter ablation: a 5-year propensity-matched analysis

**DOI:** 10.1093/europace/euaf143

**Published:** 2025-07-24

**Authors:** Issam Motairek, Chadi Tabaja, Arwa Younis, Ayman A Hussein, Mohamad Mdaihly, Adele Watfa, Wael Jaber, Pasquale Santangeli, Mina Chung, Walid I Saliba, Oussama M Wazni

**Affiliations:** Cardiac Electrophysiology and Pacing Section, Department of Cardiovascular Medicine, Cleveland Clinic, 9500 Euclid Avenue, Cleveland, OH, USA; Cardiac Electrophysiology and Pacing Section, Department of Cardiovascular Medicine, Cleveland Clinic, 9500 Euclid Avenue, Cleveland, OH, USA; Cardiac Electrophysiology and Pacing Section, Department of Cardiovascular Medicine, Cleveland Clinic, 9500 Euclid Avenue, Cleveland, OH, USA; Cardiac Electrophysiology and Pacing Section, Department of Cardiovascular Medicine, Cleveland Clinic, 9500 Euclid Avenue, Cleveland, OH, USA; Cardiac Electrophysiology and Pacing Section, Department of Cardiovascular Medicine, Cleveland Clinic, 9500 Euclid Avenue, Cleveland, OH, USA; Cardiac Electrophysiology and Pacing Section, Department of Cardiovascular Medicine, Cleveland Clinic, 9500 Euclid Avenue, Cleveland, OH, USA; Cardiac Electrophysiology and Pacing Section, Department of Cardiovascular Medicine, Cleveland Clinic, 9500 Euclid Avenue, Cleveland, OH, USA; Cardiac Electrophysiology and Pacing Section, Department of Cardiovascular Medicine, Cleveland Clinic, 9500 Euclid Avenue, Cleveland, OH, USA; Cardiac Electrophysiology and Pacing Section, Department of Cardiovascular Medicine, Cleveland Clinic, 9500 Euclid Avenue, Cleveland, OH, USA; Cardiac Electrophysiology and Pacing Section, Department of Cardiovascular Medicine, Cleveland Clinic, 9500 Euclid Avenue, Cleveland, OH, USA; Cardiac Electrophysiology and Pacing Section, Department of Cardiovascular Medicine, Cleveland Clinic, 9500 Euclid Avenue, Cleveland, OH, USA

**Keywords:** Atrial fibrillation, Left atrial appendage occlusion, Catheter ablation, Stroke prevention, Cardiovascular outcomes

Left atrial appendage occlusion (LAAO) is an established intervention for stroke prevention in patients with atrial fibrillation (AF) who are unable to tolerate long-term anticoagulation. Catheter ablation (CA) is a widely used rhythm control strategy, and in recent years, there has been increasing interest in performing both procedures concomitantly. While prior studies have explored the safety and feasibility of this approach, long-term comparative outcomes between LAAO alone and combined LAAO with ablation remain incompletely understood.

We used the TriNetX research network to conduct a retrospective analysis comparing AF patients who underwent LAAO alone (*n* = 27 576) with those who underwent both LAAO and CA (*n* = 4704). Patients in both cohorts were required to have a diagnosis of AF and procedural codes for LAAO; the combined group also required codes for CA recorded during the same or closely timed clinical encounter. To maintain a non-valvular AF population, individuals with prosthetic heart valves or mitral stenosis were excluded. Propensity score matching (1:1) was performed on 55 demographic, clinical, and medication variables, resulting in two balanced cohorts of 3264 patients each. Matching ensured alignment across key clinical factors, including mean age (74.4 ± 7.9 years), sex distribution (61.6% male), and prevalence of comorbidities such as diabetes (32.1%), chronic kidney disease (26.5%), prior stroke or transient ischemic attack (19.3%), and heart failure (47.4%).

We assessed 5-year outcomes beginning 1 day after the index procedure. The primary endpoints were ischaemic stroke, major bleeding, myocardial infarction (MI), device thrombosis, and all-cause mortality. Kaplan–Meier and Cox proportional hazards models were used to estimate outcome incidence and hazard ratios (HRs) with 95% confidence intervals (CIs).

There were no significant differences in any clinical endpoint. Ischaemic stroke occurred in 1.2% of the LAAO group and 1.1% of the combined group (HR: 1.09; 95% CI: 0.68–1.73; *P* = 0.73). Major bleeding was observed in 6.8% vs. 6.7% (HR: 1.02; 95% CI: 0.82–1.26; *P* = 0.88), MI in 3.6% in both groups (HR: 1.02; 95% CI: 0.78–1.34; *P* = 0.90), device thrombosis in 1.3% vs. 1.2% (HR: 1.02; 95% CI: 0.66–1.58; *P* = 0.91), and all-cause mortality in 5.1% of both groups (HR: 0.99; 95% CI: 0.80–1.23; *P* = 0.95) *Figure 1*. Five-year anticoagulation (AC) use remained similar between groups (∼23% in both arms), highlighting that procedural choice did not influence longer-term AC trends.

These findings align with prior research, including a recent meta-analysis of 51 802 patients, which reported no significant differences in stroke, major bleeding, or thromboembolic events between patients undergoing combined LAAO and ablation vs. LAAO alone.^1^ Similarly, a real-world propensity-matched study found comparable all-cause mortality and thromboembolic event rates at 6 months between these two approaches.^2^ Our study extends these findings by providing a more prolonged follow-up of 5 years and incorporating a larger cohort of patients.

Left atrial appendage occlusion has garnered increasing attention, particularly following randomized controlled trials demonstrating its benefits when compared to the gold standard of anticoagulation.^3^ While such studies have primarily evaluated LAAO as an alternative to pharmacologic therapy, our study focuses on procedural strategy by directly comparing standalone LAAO with combined LAAO and ablation. The results suggest that the decision to perform LAAO alone or in conjunction with ablation should be guided by individual patient characteristics rather than an expectation of differential clinical outcomes.

The integration of CA and LAAO into a single procedure has been proposed as a means to streamline AF management, potentially reducing procedural burden and healthcare utilization. Recent data from a European survey support this growing practice, highlighting increased real-world interest in combining both strategies, though LAAO is still often reserved for high-risk patients or those with complications such as stroke.^4^ Similarly, the EWOLUTION registry showed that one in six patients undergoing LAAO died within 2 years, underscoring the importance of thoughtful patient selection, particularly when combining procedures.^5^ Prior studies have demonstrated the feasibility of this approach, but our findings suggest that long-term clinical outcomes remain similar whether LAAO is performed alone or in combination with ablation.^6^

Importantly, a recent international consensus document provides practical guidance for non-implanting physicians on patient selection, timing, and referral for LAAO, reinforcing that appropriate case selection remains key when considering combined procedures.^7^ Recent feasibility data also support the technical safety and efficiency of combining pulsed field ablation with LAAO, reporting minimal additional procedural time and no major complications in carefully selected patients.^8^ While there may be logistical advantages to a combined procedure, such as reduced hospital visits and procedural sedation, these factors must be weighed against procedural complexity and patient selection.

This study has limitations. As a retrospective database analysis, it is subject to potential selection bias despite the use of propensity score matching. Additionally, data on arrhythmia burden, specific ablation techniques, recurrence following ablation, worsening of renal function,^9^ or worsening of other comorbidities, as well as occurrence of peri-device leaks^10^ and antithrombotic treatments at long term, were not available. These limitations restrict the ability to fully assess the impact of rhythm control strategy on outcomes. Future prospective randomized studies with longer-term follow-up are needed to further delineate the role of combined LAAO and ablation in AF management.

In conclusion, this 5-year analysis of a large, multi-institutional cohort demonstrates that LAAO alone and combined LAAO with ablation yield comparable outcomes with respect to stroke, major bleeding, MI, device-related thromboembolism, and mortality. These findings build upon prior research by providing the longest follow-up in this field and suggest that LAAO alone remains an effective stroke prevention strategy in AF patients. The decision to perform combined LAAO and ablation should be individualized, considering procedural logistics and patient-specific factors rather than an expectation of superior long-term outcomes.

**Figure 1:**
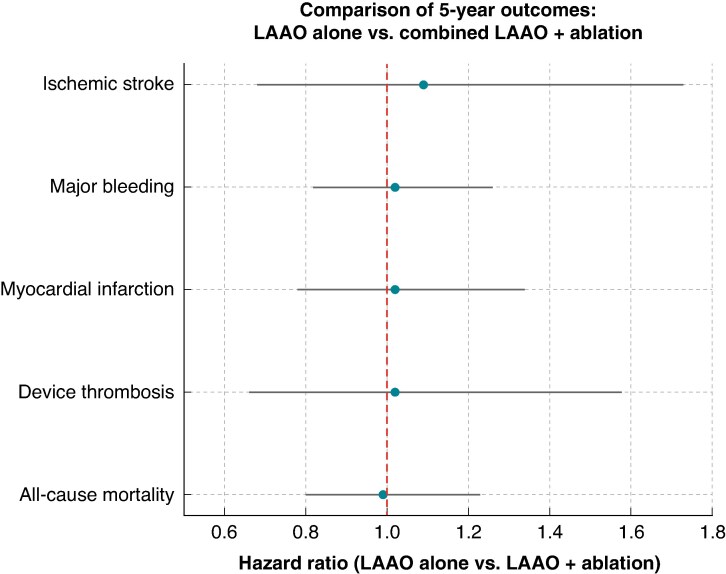
Comparison of 5-year outcomes between LAAO alone and combined LAAO + ablation. This forest plot displays 5-year clinical outcomes comparing patients who underwent LAAO alone vs. those who underwent combined LAAO and CA. Hazard ratios with 95% CIs are shown for ischaemic stroke, major bleeding, MI, device thrombosis, and all-cause mortality. Outcomes were derived from a propensity-matched cohort (*n* = 3264 per group) using the TriNetX research network. No statistically significant differences were observed between the groups across all measured outcomes.

## Data Availability

The data underlying this article were provided by TriNetX, LLC under license and cannot be shared publicly. Data will be shared on reasonable request to the corresponding author with permission of TriNetX.
